# Pharmacogenomics

**Published:** 2008-10

**069**

**Effect of antituberculosis drug therapy on hepatic drug metabolizing enzyme CYP2C9**

Melvin G, Shewade DG, Saka VK, Adithan C

**Jawaharlal Institute of Postgraduate Medical Education and Research, Pondicherry, India.**

**Aim:**

To study the effect of ATT (anti tuberculosis therapy) on induction of CYP2C9 in tuberculosis patients with polymorphic *CYP2C9* gene.

**Materials and Methods:**

111 tuberculosis patients (male - 67, female - 44) were included in the study based on the exclusion – inclusion criteria. Before commencing ATT, they were given Tab phenytoin 300 mg orally and a blood sample was collected after 3 hours. Genotyping for *CYP2C9* gene was carried out using PCR-RFLP (polymerase chain reaction-restriction fragment length polymorphism) method to detect those with polymorphic gene. Phenotyping for CYP2C9 enzyme was done in 49 patients by measuring the ratio of phenytoin and its metabolite p-HPPH (para hydroxy phenyl hydantoin) by reverse phase HPLC (high performance liquid chromatography) method before and after one month of ATT

**Results:**

The mean ratio of phenytoin/p-HPPH before ATT in CYP2C9*1*1, CYP2C9*1*2 and CYP2C9*1*3 group were 36.45 ± 14.01, 37.81 ± 19.09, 44.72 ± 16, 71 and after one month of ATT were 9.81 ± 1.85, 21.21 ± 12.00 and 11.99 ± 4.94 respectively. In CYP2C9*1*1 there was significant reduction in phenytoin metabolic ratio (*P*<0.05) by paired t test.

**Conclusion:**

ATT has been shown to cause enzyme induction of CYP2C9 in TB patients. However statistically significant induction was found only in CYP2C9*1*1 and not in the heteromutant *CYP2C9* genotype. Thus the genotype of the individual plays an important role in the degree of induction by antituberculosis treatment.

**070**

**Newer mechanistic pathways pertaining to antidepressant action of escitalopram using acute and chronic animal models of depression and underlying immunological basis of depression**

Soni S, Shah A, Agarwal R, Pandey D, Mahesh R

**Birla Institute of Technology and Science, Pilani, Rajasthan, India.**

Escitalopram (ESC), a Selective Serotonin Reuptake Inhibitor (SSRI), is an established antidepressant and anxiolytic. It increases extracellular serotonin in the brain by blocking the serotonin (5-HT) transporter (SERT). The present study was designed to explore the various mechanistic pathways of escitalopram using various acute and chronic models of depression and showing the immunological basis of depression. Acute treatment of ESC (5, 10 and 20 mg/ kg/10ml, i.p) was given to mice and were subjected to forced swim test (FST), tail suspension test (TST), reversal of reserpine (1mg/ kg) induced hypothermia, elevated plus maze, open field test, social interaction test etc. Interaction studies with 5-HT_2_ agonist (*metachlorophenylpiperazine*, mCPP) and 5-HT_2_ antagonist (ketanserin) were conducted. The chronic (14 days) effect of ESC was examined in olfactory bulbectomized (OBX) rats treated with mCPP (1mg/kg). *Macrophage hypothesis* of depression was evaluated by Differential Leucocyte Cell (DLC) count and taking the weight of spleen and thymus in OBX rats after 14 days of ESC treatment. mCPP induced immobility was decreased by ESC and ketanserin was found to potentiate the effect of ESC in FST. Chronic treatment with ESC significantly reversed the post OBX behavior in mCPP treated rats. None of the tested dose of ESC altered the locomotor activity when administered alone, which showed blockade of pharmacological actions of mCPP. Chronic treatment with ESC normalized the DLC count and spleen and thymus weight in OBX rats. In conclusion, distinct 5-HT receptor targets for ESC were found in addition to immunological basis of depression.

**071**

**Intra- and inter- ethnic variations in *GSTP1***

Dhawan D, Padh H

**B. V. Patel PERD Centre, Ahmedabad, India.**

Glutathione-S-transferases (GSTs) are crucial for cell defence system. These phase II enzymes are involved in the detoxification of a variety of chemotherapeutics including platinum through conjugation to glutathione. GSTP1, located on chromosome 11q13, is a part of the different isoenzymes of cytosolic GSTs. The GSTP1 isoform has been detected at high levels within the gastrointestinal tract. Cancer patients with higher levels of GSTP1 are shown to have less sensitivity to platinum agents. A313G SNP in exon 5 of GSTP1 leads to Ile105Val, resulting in reduced enzyme activity and therefore efficacy and/or toxicity to platinum based drugs. Since allelic and genotypic variations have been observed in different populations and ethnic groups in various parts of the world, this study aimed to establish the prevalence of GSTP1 (Ile/Val) genotypes in Indian population and compare it with other populations. Genotyping was performed using allele-specific PCR in 137 healthy Indian individuals. The genotypic distribution of GSTP1 (Ile/Val) were Ile/Ile- 45.2%, Ile/Val- 43.1% and Val/Val- 11.7%, whereas the allelic frequencies were 0.667 for Ile and 0.333 for Val allele. There was a significant variation in the observed genotypic frequencies as compared to North Indians (p ≤ 0.05) and Chinese (p ≤ 0.01). Thus, our results signify an impact of ethnicity and provide a basis for future epidemiological and clinical studies.

**072**

**Targeted medicines: Are we there yet?**

Dehar N^1^, Arora P^1^, Gupta A^2^

**^1^M.M. Institute of Medical Sciences and Research, Ambala, ^2^Government Medical College, Patiala, India.**

We are at the beginning point of the use of targeted medicines and personalized medicines. The recent inclusion of pharmacogenomics into the drug development attests to this process. There is an urgent need to change the current drug development model. As stated in the FDA critical path document, the productivity and innovation have declined over the past 10yrs; 45% decrease in advances in science not yielding many NMEs since 1996-97. One of the limitations of the current drug development process is that it is skewed to a large group of patients and not to the individual patient. So there is a need to have a different type of drug development model that is synergistic to the current model and will focus upon potential responders and nonresponders. The key is to identify those subsets of patients who react differently than the majority of the population, in relation to response or the adverse effects. So thinking about pharmacogenomics as a tool to target and identify the baseline genomic profiles, the drug development process has an opportunity to enable us to achieve that. The major drivers pushing us towards targeted medicines are: short comings of the current drug development process, rapid advances in molecular biology and disease pathophysiology, changes in regulatory expectations- heightened focus on individual benefit and risk. FDA is working to advance its capabilities and interpret genomic data. The major challenge is the general lack of the familiarity with pharmacogenomics data because the science is so new and is constantly evolving in new ways. “Incorporating pharmacogenomics into prescribing will represent a major change for health care community”

